# Systemic immune-inflammation index mediates the association between metabolic dysfunction-associated fatty liver disease and sub-clinical carotid atherosclerosis: a mediation analysis

**DOI:** 10.3389/fendo.2024.1406793

**Published:** 2024-06-18

**Authors:** Wei Wang, Xiu Li Guo, Xiu Ping Qiu, Yun Jie Yu, Mei Tu

**Affiliations:** ^1^ National Metabolic Management Center, Longyan First Affiliated Hospital of Fujian Medical University, Longyan, Fujian, China; ^2^ Fuqing City Hospital Affiliated with Fujian Medical University, Fuqin, Fujian, China

**Keywords:** systemic immune-inflammation index, metabolic dysfunction-associated fatty liver disease, sub-clinical carotid atherosclerosis, hepatic steatosis, carotid intima-media thickness

## Abstract

**Background:**

Limited research has been conducted to quantitatively assess the impact of systemic inflammation in metabolic dysfunction-associated fatty liver disease (MAFLD) and sub-clinical carotid atherosclerosis (SCAS). The systemic immune-inflammation index (SII), which integrates inflammatory cells, has emerged as a reliable measure of local immune response and systemic inflammation Therefore, this study aims to assess the mediating role of SII in the association between MAFLD and SCAS in type 2 diabetes mellitus (T2DM).

**Method:**

This study prospectively recruited 830 participants with T2DM from two centers. Unenhanced abdominal CT scans were conducted to evaluate MAFLD, while B-mode carotid ultrasonography was performed to assess SCAS. Weighted binomial logistic regression analysis and restricted cubic splines (RCS) analyses were employed to analyze the association between the SII and the risk of MAFLD and SCAS. Mediation analysis was further carried out to explore the potential mediating effect of the SII on the association between MAFLD and SCAS.

**Results:**

The prevalence of both MAFLD and SCAS significantly increased as the SII quartiles increased (*P<*0.05). MAFLD emerged as an independent factor for SCAS risk across three adjusted models, exhibiting odds ratios of 2.15 (95%CI: 1.31–3.53, *P* < 0.001). Additionally, increased SII quartiles and Ln (SII) displayed positive associations with the risk of MAFLD and SCAS (*P* < 0.05). Furthermore, a significant dose-response relationship was observed (*P* for trend <0.001). The RCS analyses revealed a linear correlation of Ln (SII) with SCAS and MAFLD risk (*P* for nonlinearity*<*0.05). Importantly, SII and ln (SII) acted as the mediators in the association between MAFLD and SCAS following adjustments for shared risk factors, demonstrating a proportion-mediated effect of 7.8% and 10.9%.

**Conclusion:**

SII was independently correlated with MAFLD and SCAS risk, while also acting as a mediator in the relationship between MAFLD and SCAS.

## Introduction

Cardiovascular disease (CVD) has emerged as a significant chronic ailment over the past century, gaining greater prominence in recent decades due to the rapid escalation of obesity and diabetes (1). Particularly concerning is its status as the primary cause of mortality among individuals with type 2 diabetes mellitus (T2DM). Subclinical carotid atherosclerosis (SCAS), a lipid-driven multifactorial inflammatory condition that manifests in the carotid artery wall, exhibits progressive thickening of the carotid intima-media thickness (cIMT) and the development of atherosclerotic plaques. Additionally, SCAS has increasingly gained recognition as an independent prognostic indicator for future cardiovascular events in T2DM (2, 3). Metabolic dysfunction-associated fatty liver disease (MAFLD) is a multi-system inflammatory disease first described in an international expert consensus in 2020, highlighting the bidirectional interplay between fatty liver and metabolic alterations (4). Remarkably, MAFLD exhibits not only an augmented susceptibility to liver-related events but also a diverse range of extrahepatic manifestations, including obesity, T2DM, and CVD (5).

Compelling evidence substantiates a substantial association between MAFLD and an elevated propensity for CVD morbidity and mortality (6, 7). The underlying mechanisms that link MAFLD to an increased risk of CVD primarily involve heightened inflammation, insulin resistance, oxidative stress, and perturbations in hepatic metabolites (8, 9). Specifically, MAFLD has garnered substantial attention for its role in promoting systemic inflammation through hepatic steatosis-secreted inflammatory proteins such as interleukin-6, C-reactive protein, fibrinogen, monocyte chemoattractant protein-1, and tumor necrosis factor-alpha (10). These inflammatory proteins augment the uptake of plasma-derived lipoproteins by macrophages, forming foam cells laden with lipids and initiating the development of atherosclerotic lesions by activating the adaptive immune system (11, 12).

The systemic immune-inflammation (SII) index integrates inflammatory cells like neutrophils, platelets, and lymphocytes, which can effectively reflect the local immune response and systemic inflammation. It was initially reported in 2014 (13) and has since been validated as an inflammatory marker in studies involving hepatic steatosis (14, 15) and atherosclerosis (16, 17). It is widely acknowledged that effective measures to quantify systemic inflammation caused by MAFLD play a pivotal role in assessing its contribution to the formation and progression of atherosclerosis toward CVD. This knowledge is essential for developing appropriate prevention and treatment strategies tailored to address the specific role of MAFLD-induced inflammation in CVD. However, to date, limited studies have quantified the role of systemic inflammation in MAFLD and SCAS. The mediating analysis is a novel research approach employed to quantify the mediating effect of a variable on the association between the independent variable and a dependent variable. This type of analysis helps understand the causal relationships between variables and the internal mechanisms underlying those relationships. Hence, this study conducted a mediation analysis to assess the mediating role of SII in the association between MAFLD and SCAS in T2DM.

## Materials and study design

### Study population

This real-world cross-sectional study utilized prospectively collected data from the National Metabolic Management Center at Longyan First Affiliated Hospital of Fujian Medical University and Fuqing City Hospital Affiliated with Fujian Medical University. A consecutive recruitment approach was employed to enroll participants with T2DM aged over 18, who were admitted between January 2023 and December 2023. Prior to their enrollment, written informed consent was diligently obtained from all participants, aligning with the ethical guidelines set forth by the Ethical Committee of Longyan First Affiliated Hospital of Fujian Medical University. Throughout the study, adherence to the principles outlined in the Declaration of Helsinki was ensured, governing the conduct of all procedures. Exclusion criteria were diligently applied to ensure the appropriate selection of participants. Specifically, individuals were excluded if they met any of the following conditions: 1. Concurrent presence of acute and chronic infections, acute diabetes complications, or experiencing acute stress states. 2. Coexistence of systemic diseases that may impede immune system function (e.g., blood, rheumatic diseases, and malignant tumors). 3. Current usage of medications known to potentially induce liver steatosis or interfere with the blood system (e.g., propylthiouracil, estrogens, tamoxifen, methimazole, and glucocorticoids). 4. Heavy alcohol consumption, defined as a daily intake of ≥ 40 g for men and ≥ 20 g for women, persisted for more than 5 years. 5. Presence of other liver comorbidities capable of inducing liver steatosis (e.g., viral hepatitis, autoimmune liver disease, or hereditary liver disease). 6. a history of cerebrovascular disease. 7. Pregnancy status or inadequate data availability. Following the recruitment process, a total of 830 participants with T2DM were included in the final analysis. Subsequently, all enrolled participants were conducted with non-enhanced CT and B-mode carotid ultrasonography to facilitate the identification and assessment of MAFLD and SCAS.

### Exposure variable assessment

MAFLD served as the exposure variable. The definition of MAFLD in T2DM adhered to the latest expert consensus statement. This definition emphasized the identification of hepatic steatosis through various methods such as imaging techniques, blood biomarkers, or liver histology (18). After enrollment, all participants underwent non-enhanced abdominal CT scans to evaluate hepatic steatosis utilizing the CT liver-spleen attenuation measurement (CT_L-S_). This CT index is specifically developed for accurately assessing hepatic steatosis (fatty liver). The CT_L-S_ was derived by dividing the mean liver attenuation by the mean spleen attenuation. A mean CTL-S value below 1.0 was indicative of hepatic steatosis. Two experienced radiologists were involved in the CTL-S measurement process to ensure consistency and reduce inter-operator variability.

### Assessment of mediator

SII is identified as the primary mediator, offering valuable insights into local immune response and systemic inflammation. SII was derived through the formula: platelet count x neutrophil count/lymphocyte count, a calculation method consistent with previous relevant research studies (13). These blood cells count was determined utilizing the Coulter LH 780 Analyzer (Beckman Coulter Ireland, Galway, Ireland).

### Outcome variable assessment

SCAS is identified as the outcome variable in this study. As established in previous reviews, SCAS was defined as the presence of cIMT exceeding 1.0mm or the presence of arterial plaque, either independently or in combination (19). Carotid artery evaluations were performed using carotid ultrasonography with an L12–5 MHz ultrasound probe, encompassing the common carotid artery, internal carotid artery, external carotid artery, and carotid bifurcation. The average intima-media thickness (IMT) of the distal vascular wall at the proximal end of the carotid artery glomus was calculated to determine the carotid intima-media thickness (cIMT). Carotid plaque was defined as a focal structure that protruded at least 0.5 mm into the arterial lumen, exhibited greater than 50% thickness compared to the surrounding IMT, or had an IMT exceeding 1.5 mm (20). All procedures were executed by skilled ultrasonologists following the standardized measurement protocol endorsed by the Society for Vascular Medicine (21).

### Study covariates

Multivariable models were constructed to adjust for the potential confounding variables that may influence the association between MAFLD and SCAS. Comprehensive demographic data, including sex, age, diabetic duration, smoking and drinking status, waist circumference (WC), systolic blood pressure (SBP), and diastolic blood pressure (DBP), was meticulously collected by trained research personnel and recorded in the information collecting system. The laboratory assessments encompassed a wide range of measurements, including serum levels of creatinine, alanine aminotransferase, aspartate aminotransferase (AST), uric acid (UA), fasting plasma glucose (FBG), serum insulin levels, triglyceride, total cholesterol (TC), low-density lipoprotein cholesterol (LDL-c), high-density lipoprotein cholesterol (HDL-c), and hemoglobin A1c (HbA1c). Biochemical parameters were conducted using an auto-biochemical analyzer (Roche Diagnostics Corporation). HbA1c levels were assessed through high-performance liquid chromatography with a D10 set (Bio-Rad). Homeostatic model assessment of insulin resistance (HOMA-IR) was calculated using the formula: fasting serum insulin (µU/ml) x FBG (mmol/l)/22.5.

### Statistical analysis

Continuous variables were reported as means ± standard deviation (SD), while discrete variables were presented as frequency tables (N, %). To assess differences among the SII quartiles, ANOVA or the K-W test was utilized for continuous variables. The chi-square or Fisher exact test was employed to compare categorical variables. The relationships between the SII, cIMT, and CT_L-S_ were assessed using Spearman correlation analysis. Subsequently, these associations underwent further scrutiny through weighted multivariable regression analyses within three distinct models. Model 1 encompassed adjustments for age, gender, diabetic duration, smoking, and drinking. In Model 2, additional adjustments were made considering cardiometabolic variables including SBP, DBP, WC, BMI, HbA1c, TG, TC, HDL-c, LDL-c, UA, and HOMA-IR. Model 3 featured supplementary adjustments for liver functional variables such as ALT and AST. Weighted logistic regression was employed to determine odds ratios (OR) and 95% confidence intervals (CI) for the correlations between SII quartiles and the risks of MAFLD and SCAS. Moreover, restricted cubic spline (RCS) analyses were carried out to explore the relationship between Ln (SII) and the risk of MAFLD and SCAS. Given the non-uniform distribution of SII data, the Ln (SII) was utilized to render the statistical analysis more appropriate. Mediation models using bootstrapping calculations were then employed to evaluate the direct impact of MAFLD on SCAS risk, as well as the indirect effect mediated by SII and Ln (SII). Finally, sensitivity analyses were performed, focusing on the associations as mentioned above in men to account for potential variations in SII categorizations and further enhance the robustness of the study findings. All statistical analyses were executed using *R* language 4.2.3 software. The significance level was set at *P* < 0.05 (two-tailed).

## Result

### Clinical characteristics of participants based on SII quartiles

A total of 830 individuals with T2DM were recruited, with 50.5% being male and an average age of 53.0 ± 8.3 years. The prevalence of MAFLD and SCAS were found to be 47.2% and 62.4%, respectively. **Table 1** provides a comprehensive overview of the clinical characteristics among participants based on SII quartiles. Significant differences were observed across the SII quartiles in variables such as WC, BMI, SBP, DBP, TG, TC, HDL-c, LDL-c, UA, HOMA-IR, cIMT, and CT_L-S_, as well as the prevalence of SCAS and MAFLD. Notably, higher SII quartiles were associated with elevated cIMT values and decreased CT_L-S_ values compared to the lower quartiles (*P* < 0.05). Moreover, there was a notable increase in the prevalence of both MAFLD and SCAS as the SII quartiles increased (*P* < 0.05).

**Table 1:** Clinical characteristics of participants according to SII quartile.

Characteristics	Overall	SII quartile				*P* value
		Q1 (<281.1)	Q2(281.1–413.6)	Q3 (413.7–552.5)	Q4 (>552.5)	
Age (year)	53.0 ± 8.3	52.9 ± 9.1	52.8 ± 7.5	53.2 ± 8.6	53.2 ± 7.9	0.253
Male, n(%)	435 (52.4)	108 (52.2)	105 (50.5)	101 (48.6)	121 (58.5)	0.205
Duration (year)	5.3 ± 3.2	5.4 ± 3.5	5.2 ± 2.9	5.1 ± 3.2	5.4 ± 3.0	0.659
BMI (kg/m2)	24.5 ± 3.0	22.3 ± 2.4	23.6 ± 2.1	25.3 ± 2.0	27.0 ± 3.1	<0.001
WC (cm)	85.8 ± 7.0	80.8 ± 4.6	83.3 ± 4.3	87.4 ± 5.6	91.8 ± 7.3	<0.001
SBP (mmHg)	133.9 ± 17.5	129.8 ± 13.0	127.9 ± 14.5	137.5 ± 10.9	140.6 ± 16.3	<0.001
DBP (mmHg)	82.1 ± 6.7	77.4 ± 6.8	78.3 ± 7.8	84.8 ± 10.4	88.1 ± 6.9	<0.001
HbA1c (%)	8.7 ± 1.1	8.5 ± 1.0	8.7 ± 0.9	8.7 ± 1.5	8.9 ± 0.9	0.089
TG (mmol/L)	2.18 ± 1.36	1.97 ± 1.45	2.26 ± 0.98	2.37 ± 1.48	2.12 ± 1.34	<0.001
TC (mmol/L)	5.19 ± 1.20	4.87 ± 1.23	5.24 ± 1.13	5.28 ± 1.17	5.39 ± 1.16	<0.001
HDL-c (mmol/L)	1.11 ± 0.25	1.17 ± 0.23	1.18 ± 0.17	1.08 ± 0.10	0.97 ± 0.11	<0.001
LDL-c (mmol/L)	3.57 ± 0.95	3.30 ± 0.96	3.63 ± 0.88	3.70 ± 0.98	3.65 ± 0.94	<0.001
UA (umol/L)	354 ± 87	324 ± 70	330 ± 72	376 ± 79	385 ± 87	<0.001
Creatinine (umol/L)	70.2 ± 13.2	70.4 ± 13.2	70.7 ± 12.5	69.3 ± 13.9	70.8 ± 13.0	0.639
ALT (IU/L)	33.9 ± 8.7	33.8 ± 9.0	34.0 ± 8.4	33.4 ± 8.5	34.3 ± 8.9	0.814
AST (IU/L)	31.8 ± 6.3	31.2 ± 5.6	32.4 ± 5.2	31.8 ± 6.1	31.7 ± 5.2	0.764
HOMA-IR	5.37 ± 2.72	4.65 ± 2.44	4.67 ± 2.26	5.86 ± 2.22	6.32 ± 2.51	<0.001
Drinking, n(%)	260 (31.3)	68 (32.9)	64 (30.8)	66 (31.7)	62 (30.0)	0.929
Smoking, n(%)	273 (32.9)	73 (35.3)	66 (31.7)	64 (30.8)	70 (33.8)	0.763
cIMT (mm)	0.96 ± 0.17	0.81 ± 0.16	0.90 ± 0.14	1.02 ± 0.11	1.10 ± 0.14	<0.001
CT_L-S_	1.05 ± 0.23	1.21 ± 0.18	1.15 ± 0.22	0.96 ± 0.14	0.85 ± 0.17	<0.001
MAFLD, n(%)	392 (47.2)	65 (31.4)	81 (38.9)	114 (54.8)	132 (63.8)	<0.001
SCAS, n(%)	518 (62.4)	94 (45.4)	114 (54.8)	141 (67.8)	169 (81.6)	<0.001

BMI, body mass index; WC, waist circumference; HbA1c, Glycated hemoglobin; UA, uric acid; TG, triglyceride; TC, total cholesterol; HDL-c, high-density lipoprotein cholesterol; LDL-c, low-density lipoprotein cholesterol; SBP, systolic blood pressure; DBP, diastolic blood pressure; HOMR-IR, homeostasis model assessment insulin resistance; cIMT, carotid intima-media thickness; MAFLD, metabolic dysfunction-associated fatty liver disease; SCAS, Sub-clinical carotid atherosclerosis; SII, systemic immune-inflammation index.

### Association between SII, cIMT, and CT_L-S_

**Figure 1:**
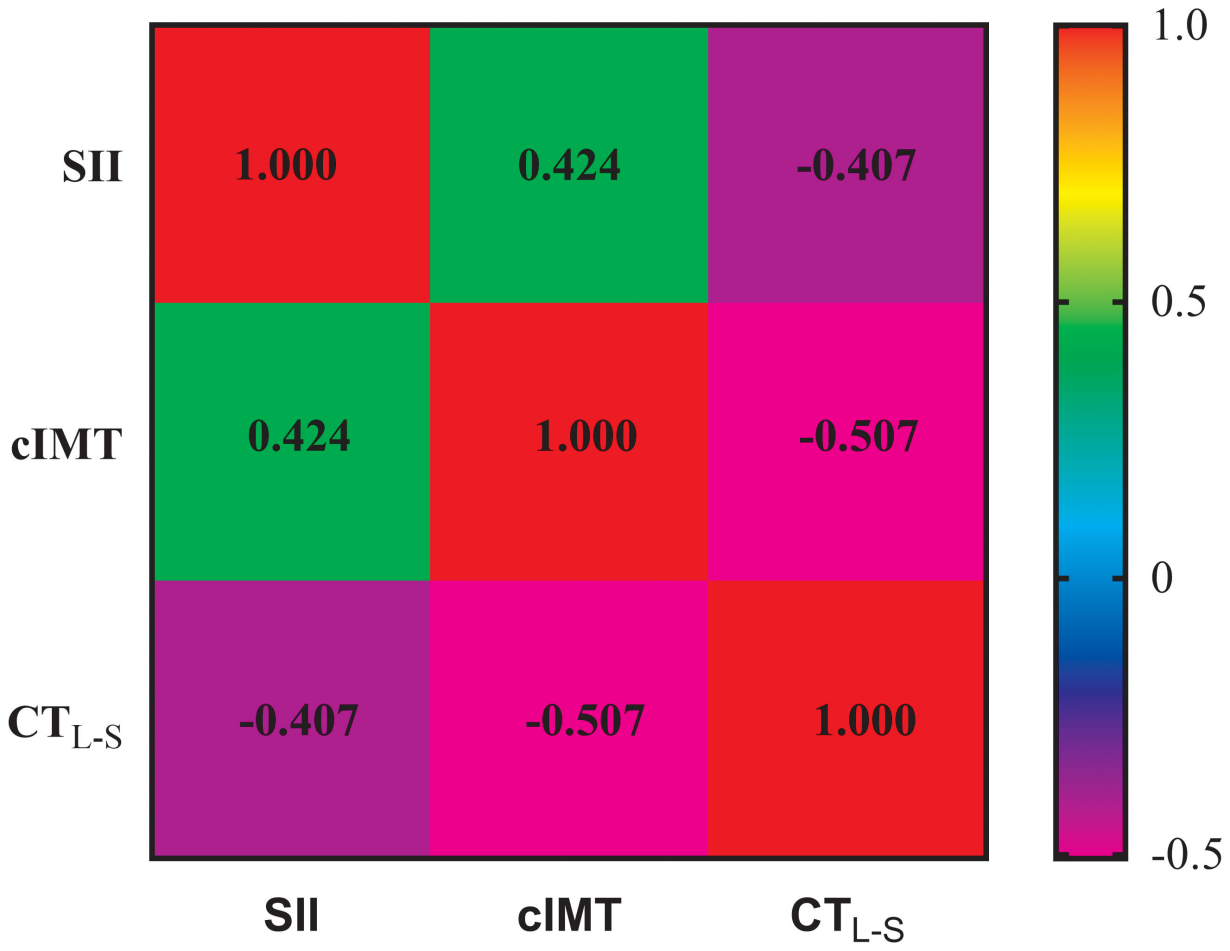
Spearman correlation analysis for the association among SII, cIMT, and CT_L-S_. SII, Systemic immune-inflammation index; cIMT, Carotid intima-media thickness; CT_L-S_, CT liver-spleen attenuation measurement.

**Figure 1** depicts the association between SII, cIMT, and CT_L-S,_ as analyzed using Spearman correlation analysis. The results showed that CT_L-S_ was negatively correlated with SII (*r*=-0.407, *P*<0.001) and CT_L-S_ (*r*=-0.507, *P*<0.001). Conversely, a positive association was observed between SII and cIMT (r=0.424, *P*<0.001). To further investigate these correlations, weighted multiple linear regression analyses were conducted. As outlined in **Table 2**. As outlined in **Table 2**, CT_L-S_ remained negatively correlated with SII (*β*=-0.147, *P*<0.001) and cIMT (*β*=-0.243, *P*<0.001) even after conducting full adjustments in Model 3. Similarly, the positive correlation between SII and cIMT persisted (*β*=0.168, *P*<0.001).

**Table 2:** Multivariate linear regression analysis for the independent associations among SII, cIMT and CT_L-S_.

Independent variables	Dependent variables	Model 1		Model 2		Model 3	
		*β*	*P* value	*β*	*P* value	*β*	*P* value
SII	cIMT	0.323	<0.001	0.189	<0.001	0.168	<0.001
SII	CT_L-S_	-0.306	<0.001	-0.159	<0.001	-0.147	<0.001
cIMT	CT_L-S_	-0.374	<0.001	-0.255	<0.001	-0.243	<0.001

Model 1: adjusted for age, gender, diabetic duration, smoking, and drinking.

Model 2: further adjustments for cardiometabolic variables, such as systolic blood pressure, diastolic blood pressure, waist circumference, body mass index, glycated hemoglobin, triglycerides, total cholesterol, high-density lipoprotein cholesterol, low-density lipoprotein cholesterol, uric acid, and homeostatic model assessment of insulin resistance.

Model 3: additional adjustments for liver functional variables like alanine aminotransferase and aspartate aminotransferase.

SII, Systemic immune-inflammation index; cIMT, Carotid intima-media thickness; CT_L-S_, CT liver-spleen attenuation measurement.

### Correlation of MAFLD with SCAS and their association with SII

**Figure 2:**
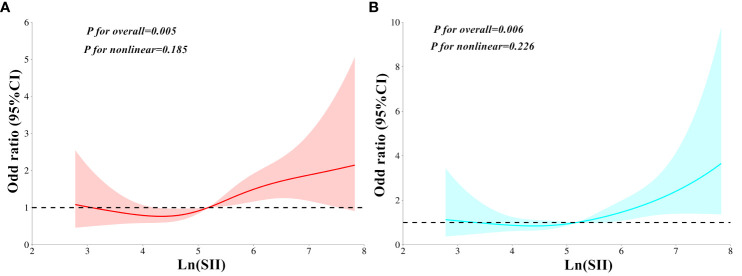
Restricted cubic spines analysis for the correlation between Ln (SII) and MAFLD risk **(A)**, as well as SCAS risk **(B)** after adjusting for Model 3. SII, Systemic immune-inflammation index; MAFLD, Metabolic dysfunction-associated fatty liver disease; SCAS, Subclinical carotid atherosclerosis. Model 3: adjusted for age, gender, diabetic duration, smoking, drinking, systolic blood pressure, diastolic blood pressure, waist circumference, body mass index, glycated hemoglobin, triglycerides, total cholesterol, high-density lipoprotein cholesterol, low-density lipoprotein cholesterol, uric acid, homeostatic model assessment of insulin resistance, alanine aminotransferase, and aspartate aminotransferase.

In all three models, MAFLD contributed an independent variable for SCAS risk. The ORs for SCAS risk were 4.79 (95%CI: 3.30–6.97, *P* < 0.001) in Model 1, 2.12 (95%CI: 1.29–3.48, *P* < 0.001) in Model 2, and 2.15 (95%CI: 1.31–3.53, *P* < 0.001) in Model 3, respectively. **Table 3** presents the findings of weighted binomial logistic regression analysis for the correlations of SII with SCAS and MAFLD risk. The results showed that higher quartiles of SII were positively associated with increased SCAS and MAFLD risk compared to the first quartile, across all three models (*P*<0.05). Meanwhile, Ln (SII) was independently correlated with increased SCAS and MAFLD risk (*P* < 0.05). In the fully adjusted Model 3, the ORs for SCAS and MAFLD risk were 2.26 (95% CI: 1.23–4.13, *P*=0.008) and 2.11 (95% CI: 1.14–3.92, *P*=0.017), respectively. Similar results were also observed when translating Ln (SII) into SII to increase by 1SD in this analysis. Additionally, a significant dose-response relationship was observed across all three models (*P* for trend <0.001). As shown in **Figure 2**, the RCS analyses demonstrated a linear correlation of Ln (SII) with SCAS and MAFLD risk in Model 3 (*P* for nonlinearity >0.05).

**Table 3:** Binomial logistic regression analysis for the correlations of SII with SCAS and MAFLD risk.

Variable	Model 1		Model 2		Model 3	
	OR (95%CI)	*P* value	OR (95%CI)	*P* value	OR (95%CI)	*P* value
SCAS						
Q1	Ref. (1.0)		Ref. (1.0)		Ref. (1.0)	
Q2	1.43(1.23–1.88)	<0.001	1.12(1.09–1.16)	<0.001	1.16(1.10–1.23)	<0.001
Q3	2.91(1.87–4.51)	<0.001	1.27(1.04–1.54)	0.018	1.20(1.09–1.32)	<0.001
Q4	3.73(1.60–8.70)	0.002	3.01(2.01–4.52)	<0.001	2.44(1.66–3.56)	0.001
Per SD increase	2.80 (2.26–3.47)	<0.001	1.94(1.39–2.71)	<0.001	1.87(1.34–2.60)	<0.001
Ln (SII)	2.54(1.75–3.69)	<0.001	2.43(1.08–5.48)	0.032	2.26(1.23–4.13)	0.008
*P* for trend	<0.001		<0.001		<0.001	
MAFLD						
Q1	Ref. (1.0)		Ref. (1.0)		Ref. (1.0)	
Q2	1.65(1.11–2.46)	0.013	1.27(1.14–1.40)	<0.001	1.13(1.07–1.21)	<0.001
Q3	2.58(1.74–3.94)	<0.001	1.96(1.22–3.16)	0.006	1.76(1.08–2.86)	0.024
Q4	4.06(3.38–4.89)	<0.001	3.26(1.43–5.52)	<0.001	2.98(1.98–4.49)	<0.001
Per SD increase	2.68(1.82–3.98)	<0.001	2.08(1.23–3.37)	0.003	1.85(1.30–2.62)	<0.001
Ln (SII)	3.82(2.14–6.87)	<0.001	2.92(1.59–5.35)	0.001	2.11(1.14–3.92)	0.017
*P* for trend	<0.001		<0.001		0.001	

Model 1: adjusted for age, gender, diabetic duration, smoking, and drinking.

Model 2: further adjustments for cardiometabolic variables, such as systolic blood pressure, diastolic blood pressure, waist circumference, body mass index, glycated hemoglobin, triglycerides, total cholesterol, high-density lipoprotein cholesterol, low-density lipoprotein cholesterol, uric acid, and homeostatic model assessment of insulin resistance.

Model 3: additional adjustments for liver functional variables like alanine aminotransferase and aspartate aminotransferase.

SII, Systemic immune-inflammation index; MAFLD, Metabolic dysfunction-associated fatty liver disease; SCAS, Subclinical carotid atherosclerosis.

### Diagnostic value of SII for MAFLD and SCAS

The diagnostic performance of SII for MAFLD and SCAS was assessed by the ROC curves analysis. The results indicate that SII exhibits a favorable diagnostic value for MAFLD and SCAS. The AUCs (95%CI) of SII for identifying MAFLD and SCAS were 0.847 (0.819–0.874) and 0.741(0.708–0.774). The optimal cut-off values of SII were 518.8 (sensitivity: 52.7%, specificity: 87.2%) for SCAS and 399.1 (sensitivity: 84.7%, specificity: 79.7%) for MAFLD.

### The mediating effect of SII and Ln (SII)

**Figure 3:**
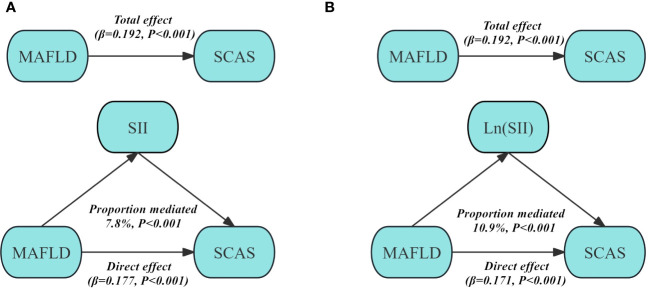
Structural model for the mediating role of SII **(A)** and Ln (SII) **(B)** in the association between MAFLD and SCAS after adjusting for Model 3. SII, Systemic immune-inflammation index; MAFLD, Metabolic dysfunction-associated fatty liver disease; SCAS, Subclinical carotid atherosclerosis. Model 3: adjusted for age, gender, diabetic duration, smoking, drinking, systolic blood pressure, diastolic blood pressure, waist circumference, body mass index, glycated hemoglobin, triglycerides, total cholesterol, high-density lipoprotein cholesterol, low-density lipoprotein cholesterol, uric acid, homeostatic model assessment of insulin resistance, alanine aminotransferase, and aspartate aminotransferase.

**Figure 3** displays the mediation analysis examining the role of SII and Ln (SII) in the association between MAFLD and SCAS. The findings indicate that a significant positive indirect effect of MAFLD associated with SCAS through SII and Ln (SII) was observed after adjustment for Model 3. The proportion mediated effect was 7.8% for SII (*P*<0.001), and 10.9% for Ln (SII) (*P*<0.001).

### Sensitivity analysis

As shown in **Supplementary Tables 1, 2**, similar results were obtained when sensitivity analysis was performed in men. The findings revealed an inverse correlation between CT_L-S_ and SII (*β*=-0.160, *P*<0.001), as well as CT_L-S_ (*β*=-0.264, *P*<0.001). Similarly, the positive correlation between SII and cIMT remained consistent (*β*=0.159, *P*<0.001). Meanwhile, Ln (SII) exhibited an independent correlation with increased SCAS and MAFLD risk (*P* < 0.05). In the fully adjusted Model 3, the ORs for SCAS and MAFLD risk were 1.79(95% CI: 1.24–2.59, *P*=0.002) and 1.35 (95% CI: 1.17–1.56, *P*<0.001), respectively. Furthermore, the proportion mediated effects of SII and Ln (SII) were 9.9% (*P*<0.001) and 13.8% (*P*<0.001) in men (**Supplementary Figure 1**).

## Discussion

This study involved prospective recruitment of participants from two centers to investigate the mediating role of SII in the association between MAFLD and SCAS in individuals with T2DM. The study made several noteworthy findings. There was a notable increase in the prevalence of both MAFLD and SCAS as the SII quartiles increased. MAFLD emerged as an independent factor for SCAS risk across three adjusted models, while increased SII quartiles and Ln (SII) displayed positive associations with the risk of MAFLD and SCAS. Additionally, SII and ln (SII) served as pivotal mediators in establishing the association between MAFLD and SCAS. Notably, sensitivity analysis unveiled consistent results in men.

MAFLD represents a systemic inflammatory liver ailment imposing substantial clinical and economic burdens due to its pronounced hepatic and extraneous ramifications like CVD (22, 23). SCAS was considered a predictor of future CVD events. This investigation unveiled that MAFLD was an independent risk factor for SCAS, presenting a heightened odds ratio of 2.15 (95% CI: 1.31–3.53). The established correlation between MAFLD and escalated SCAS risk underscores the imperative of addressing this co-occurrence within clinical realms. Recent studies emphasize the significant impact of systemic inflammation instigated by MAFLD on endothelial damage, inflammatory cell activation, and smooth muscle cell proliferation, which can directly intertwine with atherosclerosis (24, 25). Advanced methodologies like single-cell RNA sequencing and high-dimensional multi-omics have substantially enriched our understanding of immune cell subpopulation heterogeneity within the liver. These innovative approaches have shed light on emerging inflammatory mechanisms, including notable macrophage heterogeneity, involvement of auto-aggressive T cells, and the role of unconventional T cells and interactions between platelets and immune cells. These insights contribute to a deeper understanding of the complex interplay between systemic inflammation, MAFLD, and atherosclerosis (26–28). SII, a recognized measure of systemic inflammation, integrates multiple components of the immune and inflammatory systems, comprehensively assessing an individual’s overall immune-inflammatory status. This study observed that higher SII quartiles exhibited a higher prevalence of MAFLD and SCAS. This finding suggests a shared underlying background of systematic inflammation for these two conditions.

CT_L-S_ is a quantitative method employed to objectively measure the severity of hepatic steatosis, characterized by abnormal fat accumulation in the liver. On the other hand, cIMT offers valuable insights into the extent of subclinical atherosclerosis, denoting the accumulation of arterial plaque before the onset of symptoms. Xie et al. conducted a study involving 6792 adults aged 18 to 80, finding a significant positive association between SII and controlled attenuation parameters, suggesting that an increased SII may indicate more severe hepatic steatosis (29). Similarly, Song et al. analyzed data from 10505 participants and observed an independent interaction between SII and hepatic steatosis. This association was identified using weighted multivariable regression analysis and subgroup analysis (14). Additionally, Çırakoğlu et al. examined individuals with hypertension and discovered an independent correlation between SII and increased cIMT in this population (16). In line with prior research, this study showed a positive correlation between SII and cIMT, along with a negative correlation between SII and CT_L-S_ after adjusting for confounding factors using weighted multiple linear regression analyses. These results suggest that SII is linked with the severity of hepatic steatosis and cIMT. Previous studies have demonstrated a positive correlation between SII and increased risk of NAFLD and CVD mortality. For instance, Liu et al. revealed a significant positive association observed between ln (SII) and NAFLD risk (OR=1.46, 95% CI: 1.27–1.69, *P <*0.001), establishing a linear relationship between ln (SII) and NAFLD in a cohort of 10,821 adults from six cycles of the NHANES (30). Several large cohort studies have demonstrated that an elevated SII is independently associated with an increased risk of CVD mortality (31–33). Our study found similar results to the above studies. In addition, this study also conducted a sensitivity analysis in subgroups of men to account for potential variations in SII categorizations and further enhance the robustness of the study findings. As expected, the positive correlation of increased SII quartiles and Ln (SII) with the risk of MAFLD and SCAS persisted. Additionally, SII has been validated as a diagnostic index for various inflammatory digestive diseases (34, 35) and coronary artery disease (36). Similarly, this study also found that SII exhibited a favorable diagnostic value for MAFLD and SCAS. Previous studies have demonstrated that SII played a mediating role in chronic inflammatory diseases associated with CVD (37) and metabolic syndrome (38). T2DM often exhibits abnormal glucose metabolism, dysfunctional fat accumulation, insulin resistance, and dyslipidemia, all contributing to systemic inflammation and SCAS. Consequently, determining the exact impact of inflammation triggered by MAFLD on the increased risk of SCAS in individuals with T2DM is intricate due to shared risk factors between the conditions. Despite adjustment for the shared risk factors attenuating the proportion of the mediated effect related to SII and Ln (SII), its mediating role in this association remained statistically significant.

This study has several notable strengths, particularly its novel investigation into the mediating role of the SII in the relationship between MAFLD and SCAS, as well as the enrollment of a study population from two centers, which enhances the reliability and external validity of the findings. Nonetheless, it is imperative to recognize certain limitations inherent in this investigation. Firstly, the data utilized in this analysis were cross-sectional, limiting our ability to establish causality or assess temporal relationships. Secondly, despite adjusting for various known risk factors, there is still the possibility of residual confounding or unmeasured variables that could influence the interpretation of the result. Lastly, the assessment of hepatic steatosis in this study relied on unenhanced CT scans rather than the gold standard criterion of liver biopsy. This methodological variation introduces the potential for discrepancies in the accuracy of diagnosis.

## Conclusion

In conclusion, increased SII quartiles and Ln (SII) demonstrated positive associations with the risk of MAFLD and SCAS. SII and Ln (SII) partially mediated the effect of MAFLD on SCAS. These findings emphasize the impact of MAFLD on increased risk of SCAS may be achieved in part by promoting systemic inflammation. Further research is needed to validate our results and elucidate the underlying mechanisms.

## Data availability statement

The original contributions presented in the study are included in the article/**Supplementary Material**. Further inquiries can be directed to the corresponding authors.

## Ethics statement

The studies involving humans were approved by Ethical Committee of Longyan First Affiliated Hospital of Fujian Medical University. The studies were conducted in accordance with the local legislation and institutional requirements. The participants provided their written informed consent to participate in this study. Written informed consent was obtained from the individual(s) for the publication of any potentially identifiable images or data included in this article.

## Author contributions

WW: Data curation, Formal analysis, Investigation, Methodology, Writing – original draft. XG: Data curation, Formal analysis, Investigation, Writing – original draft. XQ: Data curation, Investigation, Writing – original draft. YY: Formal analysis, Investigation, Methodology, Validation, Writing – review & editing. MT: Conceptualization, Formal analysis, Investigation, Methodology, Validation, Writing – review & editing.

## Supplementary material

Supplementary Figure 1 Structural model for the mediating role of SII and Ln (SII) in the association between MAFLD and SCAS after adjusting for Model 3 in men.
