# Supplementary material for: Systemic immune-inflammation index mediates the association between metabolic dysfunction-associated fatty liver disease and sub-clinical carotid atherosclerosis: a mediation analysis
Source: Front Endocrinol (Lausanne). 2024 Jun 18;15:1406793. doi: 10.3389/fendo.2024.1406793 (PMC11217321; doi:10.3389/fendo.2024.1406793)
Supplement: Supplementary file 1 [file Table_1.docx]

| **Supplementary Table 1.** Multivariate linear regression analysis for the independent associations among SII, cIMT and CT_L-S_ in men. | | | | | | | |
| --- | --- | --- | --- | --- | --- | --- | --- |
| Independent  variables | Dependent  variables | Model 1 | | Model 2 | | Model 3 | |
|  |  | *β* | *P* | *β* | *P* | *β* | *P* |
| SII | cIMT | 0.381 | <0.001 | 0.188 | <0.001 | 0.159 | <0.001 |
| SII | CT_L-S_ | -0.393 | <0.001 | -0.195 | <0.001 | -0.160 | <0.001 |
| cIMT | CT_L-S_ | -0.410 | <0.001 | -0.275 | <0.001 | -0.264 | <0.001 |
| Model 1: adjusted for age, diabetic duration, smoking, and drinking.  Model 2: further adjustments for cardiometabolic variables, such as systolic blood pressure, diastolic blood pressure, waist circumference, body mass index, glycated hemoglobin, triglycerides, total cholesterol, high-density lipoprotein cholesterol, low-density lipoprotein cholesterol, uric acid, and homeostatic model assessment of insulin resistance.  Model 3: additional adjustments for liver functional variables like alanine aminotransferase and aspartate aminotransferase.  SII: Systemic immune-inflammation index. cIMT: Carotid intima-media thickness. CT_L-S_: CT liver-spleen attenuation measurement. | | | | | | | |

| **Supplementary Table 2.** Binomial logistic regression analysis for the correlations of SII with SCAS and MAFLD risk in men. | | | | | | |
| --- | --- | --- | --- | --- | --- | --- |
| Variable | Model 1 | | Model 2 | | Model 3 | |
|  | OR (95%CI) | *P* | OR (95%CI) | *P* | OR (95%CI) | *P* |
| SCAS | | | | | | |
| Per SD increase | 3.21(2.29-4.45) | <0.001 | 2.03(1.23-3.35) | 0.006 | 1.46(1.06-2.01) | 0.022 |
| Ln (SII) | 3.17(1.92-5.24) | <0.001 | 2.76(1.21-6.31) | 0.026 | 1.79(1.24-2.59) | 0.002 |
| *P* for trend | <0.001 | | <0.001 | | <0.001 | |
| MAFLD | | | | | | |
| Per SD increase | 4.33(2.66-7.04) | <0.001 | 1.98(1.30-3.03) | 0.001 | 1.74(1.56-1.94) | <0.001 |
| Ln (SII) | 2.17(1.43-3.31) | <0.001 | 1.43(1.26-1.61) | <0.001 | 1.35(1.17-1.56) | <0.001 |
| *P* for trend | <0.001 | | <0.001 | | 0.001 | |
| Model 1: adjusted for age, diabetic duration, smoking, and drinking.  Model 2: further adjustments for cardiometabolic variables, such as systolic blood pressure, diastolic blood pressure, waist circumference, body mass index, glycated hemoglobin, triglycerides, total cholesterol, high-density lipoprotein cholesterol, low-density lipoprotein cholesterol, uric acid, and homeostatic model assessment of insulin resistance.  Model 3: additional adjustments for liver functional variables like alanine aminotransferase and aspartate aminotransferase.  SII: Systemic immune-inflammation index. MAFLD: Metabolic dysfunction-associated fatty liver disease. SCAS: Subclinical carotid atherosclerosis. | | | | | | |
