# Supplementary figures and images for: Systemic immune-inflammation index mediates the association between metabolic dysfunction-associated fatty liver disease and sub-clinical carotid atherosclerosis: a mediation analysis
Source: Front Endocrinol (Lausanne). 2024 Jun 18;15:1406793. doi: 10.3389/fendo.2024.1406793 (PMC11217321; doi:10.3389/fendo.2024.1406793)

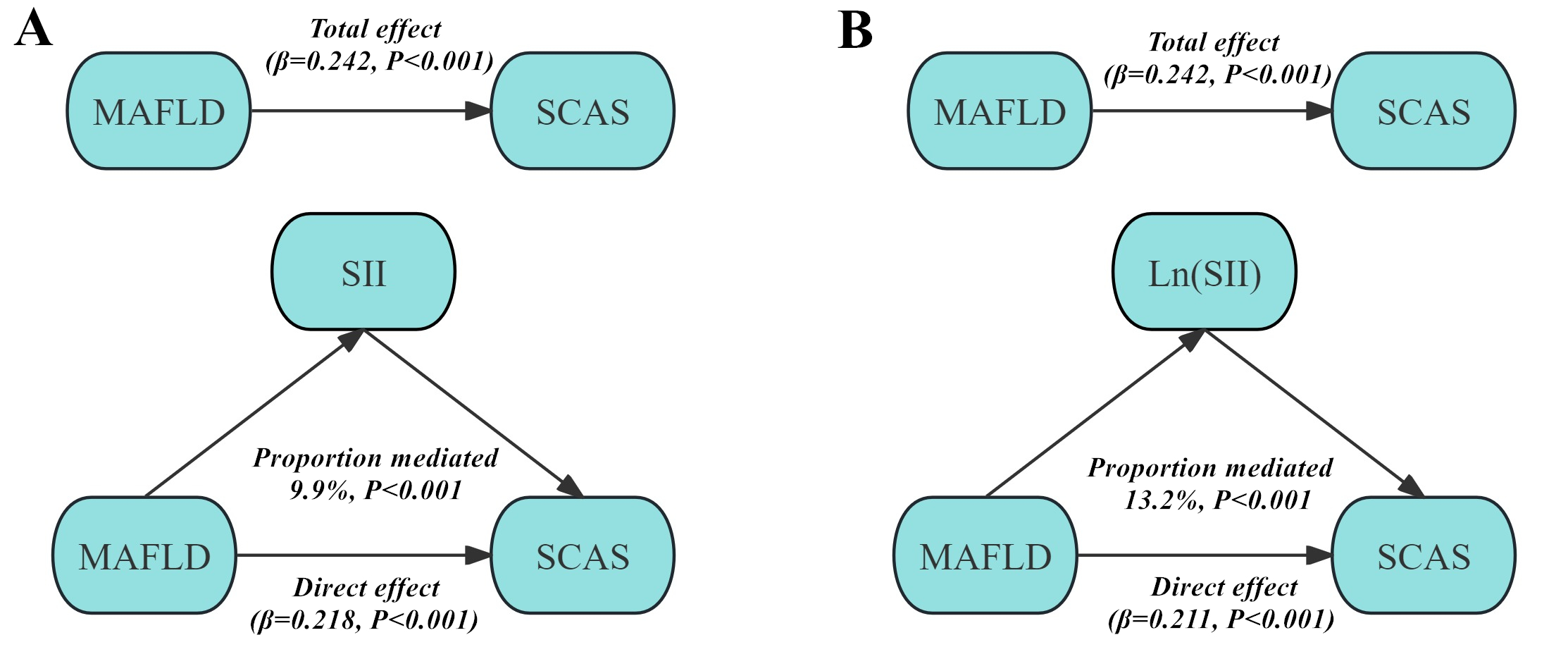

Supplement: Supplementary Figure 1 — Structural model for the mediating role of SII and Ln (SII) in the association between MAFLD and SCAS after adjusting for Model 3 in men. [file Image_1.jpeg]
